# Smooth Pursuit Eye Movements in Children with Strabismus and in Children with Vergence Deficits

**DOI:** 10.1371/journal.pone.0083972

**Published:** 2013-12-20

**Authors:** Cynthia Lions, Emmanuel Bui-Quoc, Sylvette Wiener-Vacher, Magali Seassau, Maria Pia Bucci

**Affiliations:** 1 Unité Mixte de Recherche 676 Inserm - Université Paris Diderot, Hôpital Robert Debré, Paris, France; 2 Service d'Ophtalmologie, Hôpital Robert Debré, Paris, France; 3 Vestibular and Oculomotor Evaluation Unit, ORL Department, Robert Debré Hospital, Paris, France; 4 e(ye)BRAIN, Ivry-sur-Seine, France; University of Leicester, United Kingdom

## Abstract

**Purpose:**

The objective of our study was to examine horizontal smooth pursuit performance in strabismic children and in children with vergence deficits, and to compare these data with those recorded in a group of control age-matched children.

**Methods:**

Binocular eye movements were recorded by video-oculography in ten strabismic children (mean age: 9.8±0.8) and seven children with vergence deficits (mean age: 10.8±0.6). Data were compared to that of age-matched control children (mean age: 9.8±0.8 years).

**Results:**

Catch-up saccades amplitude in strabismic children and in children with vergence deficits were significantly higher than in control age-matched children. Moreover, in strabismic children the amplitude of catch-up saccades was significantly higher in rightward than in leftward direction. The number of catch-up saccades was also significantly higher in rightward than in leftward direction. The gain value of pursuits in rightward direction was significantly higher in the right eye than in the left one; for the right eye, the gain value was significantly higher in rightward than in leftward direction. Binocular coordination of pursuit was better in control age-matched children than in children with vergence deficits and than in strabismic children.

**Conclusions:**

Binocular coordination of pursuit is abnormal in children with vergence deficits and worse in strabismic children. Binocular vision plays an important role in improving binocular coordination of pursuit.

## Introduction

Smooth pursuits are slow conjugate movements which maintain the image of a moving object close to the fovea, the area of highest acuity on the retina. To maintain the moving object's image on the fovea, eye velocity must correspond as much as possible to the speed of the moving object. Frequently, the eyes are not able to keep up with the motion of the object, and as the visual system senses the positional error created, it realigns the eyes, normally with very rapid saccadic movements, *i.e.* catch-up saccades[1].

Smooth pursuits are movements which can be explored as early as at two months of age [2], [3] even if at that age catch-up saccades are very frequent and the smooth pursuit gain (*i.e.* the ratio of the smooth pursuit eye velocity to target velocity) is very low (about <0.6). With age, pursuit gain improves and the number of catch-up saccades decreases significantly, reaching adult values during adolescence [2], [4]. These authors suggest that the improvement in smooth pursuits reported during childhood and teenagehood is related to the maturation of brain myelination, which progresses from dorsal to ventral brain regions [5].

No study dealing with smooth pursuit performances in children with poor vergence control (*i.e.* with vergence abnormalities) or with strabismus exists. Both children with vergence abnormalities and strabismic children have poor visual disparity vergence capability, and strabismic population can also have poor or absent binocular vision.

Previous studies from our group showed that about 7% of children consulting the Ear Nose Throat (ENT) department of our hospital for vertigo headaches and equilibrium disorders showed normal vestibular function but presented signs of vergence abnormalities assessed by orthoptic tests [6]. This population of children also showed deficits in the latency and accuracy of saccades and vergence eye movements [7], and a recent study from Gaertner *et al*. [8] reported poor interaction between the saccade and vergence subsystems leading to poor quality in the binocular coordination of saccades when reading a text. Similar results have also been reported in children with strabismus [7], [9]. We hypothesized that such poor binocular control could be due to impairment in the central structures related to sensory disparity inputs occurring in strabismic children.

In the literature, several studies have been conducted on smooth pursuits in adult subjects with strabismus. Schor *et al.* [10] reported a marked directional impairment of pursuits of a target moving towards the nasal direction in amblyopic adults with constant strabismic amblyopia. Ciuffreda *et al.* [11] also reported in adult subjects with strabismic amblyopia a large number of catch-up saccades (saccades with short amplitudes in the direction of the moving target) during pursuit movements when the amblyopic eye was viewing; in contrast, pursuits showed normal gain when subjects were viewing with their dominant eye. For these authors, such high frequency of catch-up saccades could be related to the presence of amblyopia rather than strabismus.

Tychsen *et al.* [12] found in a preliminary study an asymmetry in pursuit direction in fourteen adults with early onset strabismus. During monocular viewing, pursuits were impaired when the target moved temporally and they were normal when the target moved in thee temporal-to-nasal direction. These results have been confirmed subsequently in seven adults with strabismus without binocular vision [13]. Strabismic subjects showed naso-temporal asymmetries, such that nasally-directed target motion evoked more vigorous pursuits.

Bedell *et al.* [14] examined pursuits in eleven young adults with strabismic amblyopia, and they found that the gain of pursuits in the nasal direction was better than that in the temporal direction. This asymmetry was more pronounced in the amblyopic eye than in the non-amblyopic eye. Recently, Nawrot *et al.* [15] studied the role of smooth pursuit in the perception of depth from motion parallax in seven esotropic subjects. They found a correlation between gain value and perception of depth from motion parallax. When gain of pursuit was normal, the perception of depth from motion parallax was also correct. On the other hand, when esotropic subjects showed poor pursuit gain from the amblyopic eye, they had difficulty with the perception of depth from motion parallax. Such relationship between pursuit and motion parallax suggests the role of extra-retinal signal in neural circuits controlling the perception of depth from motion parallax.

The objective of our study was to examine horizontal smooth pursuit performance in children with vergence deficits and in children with strabismus, and to compare these data with those recorded in a group of control age-matched children. In order to further explore the role of the vergence system and of binocular vision and their relationship with pursuit system, we also measured the quality of binocular coordination during pursuit eye movements. Our driving hypothesis, based on previous studies from our research group, is that impaired vergence disparity inputs and poor binocular sensory capabilities could cause poor binocular motor pursuit control.

## Materials and Methods

### Subjects

Ten strabismic children between 6.8 and 13.8 years old (mean age: 9.8±0.8) and seven children with vergence deficits between 8.2 and 12.8 years old (mean age: 10.8±0.6) participated in the study. Strabismic children and children with vergence deficits were recruited from the department of Ophthalmology and of ENT of Robert Debré children hospital in Paris. A group of ten matched control children (mean age: 9.8±0.8 years) were also tested.

The investigation adhered to the principles of the Declaration of Helsinki and was approved by our institutional Human Experimentation Committee (Comité de Protection des Personnes CPP Ile de France V, Hôpital Saint Antoine). Informed parental consent was obtained for each subject after the nature of the procedure had been explained. Children given verbal consent and they were advised that could stop the experiment if and when they wanted to.

### Ophthalmological and orthoptic examination

All children underwent ophthalmological and orthoptic examination to evaluate their visual functions. Clinical data of the two groups of children are shown in Table 1 and Table 2. The visual acuity was measured for each eye separately at far (5 m) with the Monoyer chart (an optometric chart containing 10 rows of letters, each row corresponding to 1/10 visual acuity). Heterophoria (*i.e.* the latent deviation of one covered eye when the other is not covered) was measured at near distance (30 cm) by using the cover-uncover test. Heterotropia (*i.e.* the manifest deviation of one eye) was measured at near and far distance by using the cover-uncover test. Measurement of convergence and divergence fusional amplitude was done at near distance using a base-in and a base-out prism bar. Near point of convergence (NPC) was also examined by placing a small accommodative target at 30 cm in the midplane in front of the child and moving it slowly towards the eye until one eye lost fixation. Stereoacuity threshold based on disparity detection was evaluated with the TNO random dot test for stereoscopic depth discrimination.

**Table 1:** Clinical characteristics of strabismic children.

Children (years)	Glasses correction	Corrected visual acuity	Angle of strabismus	Stereoacuity	Type of strabismus
			(prism D)	(TNO)	
C1 (7.7)	RE: −1.25 (−0.75) 55°	RE: 20/20	50 X′-X′T	40″	Intermittent exotropia
	LE: −1.50 (−1.00) 150°	LE: 20/20	30 X-XT		
C2 (9.2)	RE: +6.5	RE: 20/20	10 X′-X′T	40″	Intermittent exotropia
	LE: +7.5	LE: 20/20	25 X-XT		
C3 (10.4)	RE: −1.00	RE: 20/20	35 X′-X′T	60″	Intermittent exotropia
	LE: −1.00	LE: 20/20	35 X-XT		
C4 (13.7)	RE: +0.25	RE: 20/20	30 E′-E′T	240″	Acquired esotropia
	LE: +0.25 (−0.25)180°	LE: 20/20	8 E-ET		
C5 (7.1)	RE: +2.25	RE: 20/20	35 E′T	—	Acquired esotropia
	LE: +5.75	LE: 20/20	30 ET		
C6 (6.8)	RE: +1.75 (−1.00)165°	RE: 20/20	25 E′T	—	Early onset esotropia
	LE: +0.75 (−1.00)165°	LE: 20/20	25 ET		
C7 (7.3)	RE: +6.75 (−2.00)15°	RE: 20/20	45 E′T	—	Early onset esotropia
	LE: +7.25 (−2.00) 10°	LE: 20/20	35ET		
C8 (9.4)	RE: +0.5 (−0.5)100°	RE: 20/20	30 E′T	—	Early onset esotropia
	LE: 0.00 (−0.5)120°	LE: 20/20	35 ET		
C9 (11.7)	RE: −0.75 (−0.75)175°	RE: 20/20	35 X′T	—	Constant exotropia
	LE: −2.50	LE: 20/20	45 XT		
C10 (13.8)	RE: 0.00	RE: 20/20	45 E′T	—	Early onset esotropia
	LE: 0.00	LE: 20/20	45 ET		

LE, RE, left eye, right eye. The deviation of the eyes was assessed with cover-uncover test and prism; the binocular vision was evaluated with the TNO test for stereoscopic depth discrimination. X-XT  =  intermittent exotropia measured at far distance (5 m); X′-X′T  =  intermittent exotropia measured at near distance (30 cm); E-ET  =  accommodative esotropia measured at far distance (5 m); E′-E′T  =  accommodative esotropia measured at near distance (30 cm); E′T and ET, esotropia measured at near (30 cm) and at far (5 m) distance, respectively; H′TD and HTD, hypertropia of right eye measured at near (30 cm) and at far (5 m) distance, respectively.

**Table 2:** Clinical characteristics of children with vergence abnormalities.

Children	Glasses correction	Corrected visual acuity	Near Point of Convergence	Heterophoria near	Divergence near	Convergence near	Stereoacuity
(years)			(cm)	(dioptries)	(dioptries)	(dioptries)	(TNO)
C1 (8.2)	RE: +1.00	RE: 20/20	2	−2	14	25	60″
	LE: +0.75	LE: 20/20					
C2 (9.4)	RE: (−0.50)45°	RE: 20/20	8	−10	20	20	120″
	LE: (−1.50)10°	LE: 20/20					
C3 (9.9)	RE: +3.50	RE: 20/20	4	4	18	40	120″
	LE: +1.00	LE: 20/20					
C4 (11.7)	RE: +1.25	RE: 20/20	1	−4	16	20	60″
	LE: +1.25	LE: 20/20					
C5 (12)	RE: 0.00	RE: 20/20	0	6	10	45	60″
	LE: 0.00	LE: 20/20					
C6 (12.2)	RE: (−0.50)40°	RE: 20/20	9	−4	20	25	60″
	LE: (−0.75)150°	LE: 20/20					
C7 (12.8)	RE: 0.00	RE: 20/20	5	−6	2	40	60″
	LE: 0.00	LE: 20/20					

LE, RE, left eye, right eye. The deviation of the eyes was assessed with cover-uncover test and prism; the binocular vision was evaluated with the TNO test for stereoscopic depth discrimination. Negative values represent a divergent deviation, positive values a convergent deviation.

With respect to the group of children with strabismus, three children had intermittent exotropia with binocular vision in normal range (≤60 seconds of arc with the TNO test). Two children had acquired esotropia (i.e. esotropia which began after the age of 2 years old), one (C4) with binocular vision of 240″ of arc and one (C5) with no binocular vision. The other five children had early onset esotropia (i.e. which began before the age of 2 years old) with no binocular vision.

All children with vergence deficits suffered from vertigo and headaches. Two (C1 and C4) had both convergence and divergence insufficiency; three (C2, C6 and C7) had only convergence insufficiency and two children (C3 and C5) had only divergence insufficiency.

Note that in the literature, normative data for orthoptic examination varied greatly [16]–[18]. Control age-matched children were selected as normal when they did not complain of any vertigo and/or headaches, and showed normal values at the orthoptic evaluation. Orthoptic values are shown for these children in Table 3. We based our criteria on our normal orthoptic values calculated in our clinical research centre on a population of 81 subjects aged from 5 to 17 years (mean age 9.6±3 years) presenting no vertigo, no headaches, no vestibular pathology and no neurological or ophthalmologic pathology. Based on these criteria, significant differences in orthoptic examination between control and children with vergence abnormality were observed for the near point of convergence (NPC, evaluated with a small accommodative target) and the convergence fusional amplitude.

**Table 3:** Clinical characteristics of control age-matched children.

Children	Corrected visual acuity	Near Point of Convergence	Heterophoria near	Divergence near	Convergence near	Stereoacuity
(years)		(cm)	(dioptries)	(dioptries)	(dioptries)	(TNO)
C1 (7.7)	RE: 20/20	3	−2	14	35	60″
	LE: 20/20					
C2 (9.4)	RE: 20/20	5	−4	14	30	60″
	LE: 20/20					
C3 (6.8)	RE: 20/20	0	2	10	35	30″
	LE: 20/20					
C4 (10.4)	RE: 20/20	0	−2	20	40	30″
	LE: 20/20					
C5 (13.7)	RE: 20/20	5	−6	18	25	60″
	LE: 20/20					
C6 (8.1)	RE: 20/20	8	−2	12	40	60″
	LE: 20/20					
C7 (7.3)	RE: 20/20	0	2	12	30	30″
	LE: 20/20					
C8 (11.7)	RE: 20/20	0	0	16	40	60″
	LE: 20/20					
C9 (13.8)	RE: 20/20	0	−2	14	40	30″
	LE: 20/20					
C10 (9.2)	RE: 20/20	5	−8	18	30	30″
	LE: 20/20					

LE, RE, left eye, right eye. The deviation of the eyes was assessed with cover-uncover test and prism; the binocular vision was evaluated with the TNO test for stereoscopic depth discrimination. Negative values represent a divergent deviation, positive values a convergent deviation.

### Smooth pursuit

The pursuit task requires participants to follow a slowly moving visual target. Stimuli were displayed on a 22′ computer monitor. A red target of circular shape (approximately 0.5° of visual angle) was presented on a black background. Participants sat on a chair at a distance of 60 cm from the monitor. Head movements were minimized using a chin rest. Testing took place in a quiet, darkened room. A calibration task was carried out before each task.

Smooth pursuit. The circular target waveform was used twice with the same velocity (15°/s). The target was initially placed in the central position (0°) and then moved horizontally to one side until it reached the +/− 20° location, where it reversed abruptly and moved to the opposite side. A total of nine cycles were run and included in the analysis. Participants were instructed to keep their eyes on the target, wherever it moved. After a short break, children had to perform the same test again.

### Eye movement recording

Eye movements were recorded by a non-invasive system using infrared camera and mirror to record horizontal and vertical eye position independently and simultaneously for each eye: the Mobile EyeBrain Tracker (Mobile EBT®, e(ye)BRAIN, www.eye-brain.com). This eye tracker is a medical device EC-marked for medical purposes (European Community markage). Recording frequency was set to 300 Hz. The mobile eBT is linear and the precision of the system is 0.25° during static acquisition. Calibration was done at the beginning of each eye movement recordings. Calibration was done under binocular viewing. A previous study from Bucci et al. (2002) confirmed that for strabismic children both types of calibration monocular and binocular viewing were valid. During the calibration procedure, children were asked to fixate a grid of 13 points (diameter 0.5 deg) mapping the screen. Point positions in degree in the horizontal/vertical plans were: −20.9°/12.2°; 0°/12.2°; 20.9°/12.2°; −10.8°/6.2°; 10.8°/6.2°; −20.9°/0°; 0°/0°; 20.9°/0°; −10.8°/−6.2°; 10.8/−6.2°; −20.9°/−12.2°; 0°/−12.2°; 20.9°/−12.2°. Each calibration point required a fixation of 250 ms to be validated. A polynomial function with five parameters was used to fit the calibration data and to determine the visual angles. Calibration factors for each eye were determined from the eye positions during the calibration procedure; it should be >0.8 (see Lions *et al.* [9]). There is no obstruction of the visual field with the recording system and the calibrated zone covers a visual angle of ±22°.

### Procedure

Children were seated on a chair in a dark room, in front of a flat screen displaying the target stimulating smooth pursuit eye movements. The head of the child was held straight with a head-rest; viewing was binocular.

For each pursuit task (lasting a couple of minutes) a calibration procedure was presented to the child. The duration of the task was kept short to avoid head movements.

### Data analysis

Calibration factors for each eye were determined from the eye positions during the calibration procedure (see Lions *et al.* 2013). The software MeyeAnalysis (provided with the eye tracker, e(ye)BRAIN) was used to extract pursuit eye movements from the data.

Detection of saccades during pursuit was based on criteria of minimum amplitude (2°) and velocity (30°/s). Catch-up saccades were defined as saccades in the target direction that serve to reduce position error and to bring the eye closer to the target. The number of catch-up saccades was counted for each cycle. Amplitude was measured for each catch-up saccade. Pursuit gain was obtained by dividing eye velocity by target velocity for each cycle. Scores were then averaged across cycles for each test. Disconjugacy was obtained by changing vergence between the beginning and the end of the pursuit.

Data were entered in a repeated measures ANOVA test with the three groups of children (children with strabismus, children with vergence deficits and control age-matched children) as inter-subject factor, and the direction of smooth pursuit movement or the eye performing the movement as main-factors. We performed this analysis for the following parameters: individual means of their catch-up saccade's amplitude, number of catch-up saccades, gain value, and disconjugacy between the two eyes measured during the pursuit movement. The post hoc analysis was done with the Fisher LSD post hoc test. The effect was considered significant when the p-value was below 0.05.

## Results

### Amplitude of the catch-up saccades

**Figure 1:**
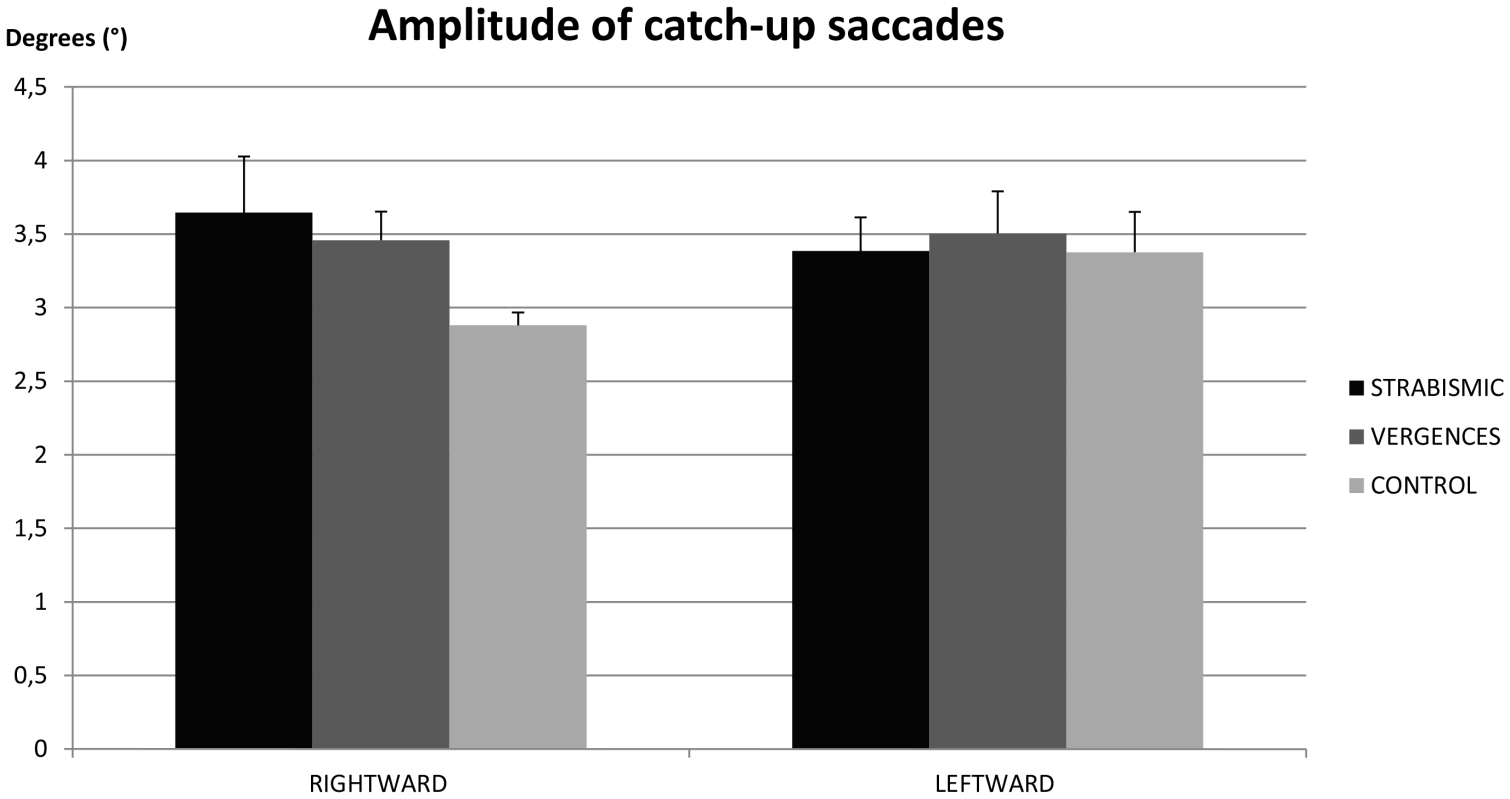
Amplitude of the catch-up saccades. Individual mean of the amplitude of the catch-up saccades for strabismic children, children with vergence deficits and control age-matched children. Vertical bars indicate the standard error.

Figure 1 shows the mean amplitude of catch-up saccades in strabismic children, in children with vergence deficits and in control age-matched children. The ANOVA test showed a significant interaction between children and direction (F_(2.21)_ = 6.72, P<0.005). Post hoc comparison showed that amplitude of saccades in rightward direction for strabismic children was significantly higher than for non-strabismic children (p<0.002). In strabismic children, amplitude of catch-up saccades in rightward direction was significantly higher than in leftward direction (p<0.007). In children with vergence deficits the amplitude of catch-up saccades in rightward direction was significantly higher than that measured in non-strabismic children (p<0.05). There was no significant difference between rightward and leftward directions. In non-strabismic children, amplitude of catch-up saccades was significantly smaller in rightward direction than in leftward direction (p<0.04).

### Number of catch-up saccades

**Figure 2:**
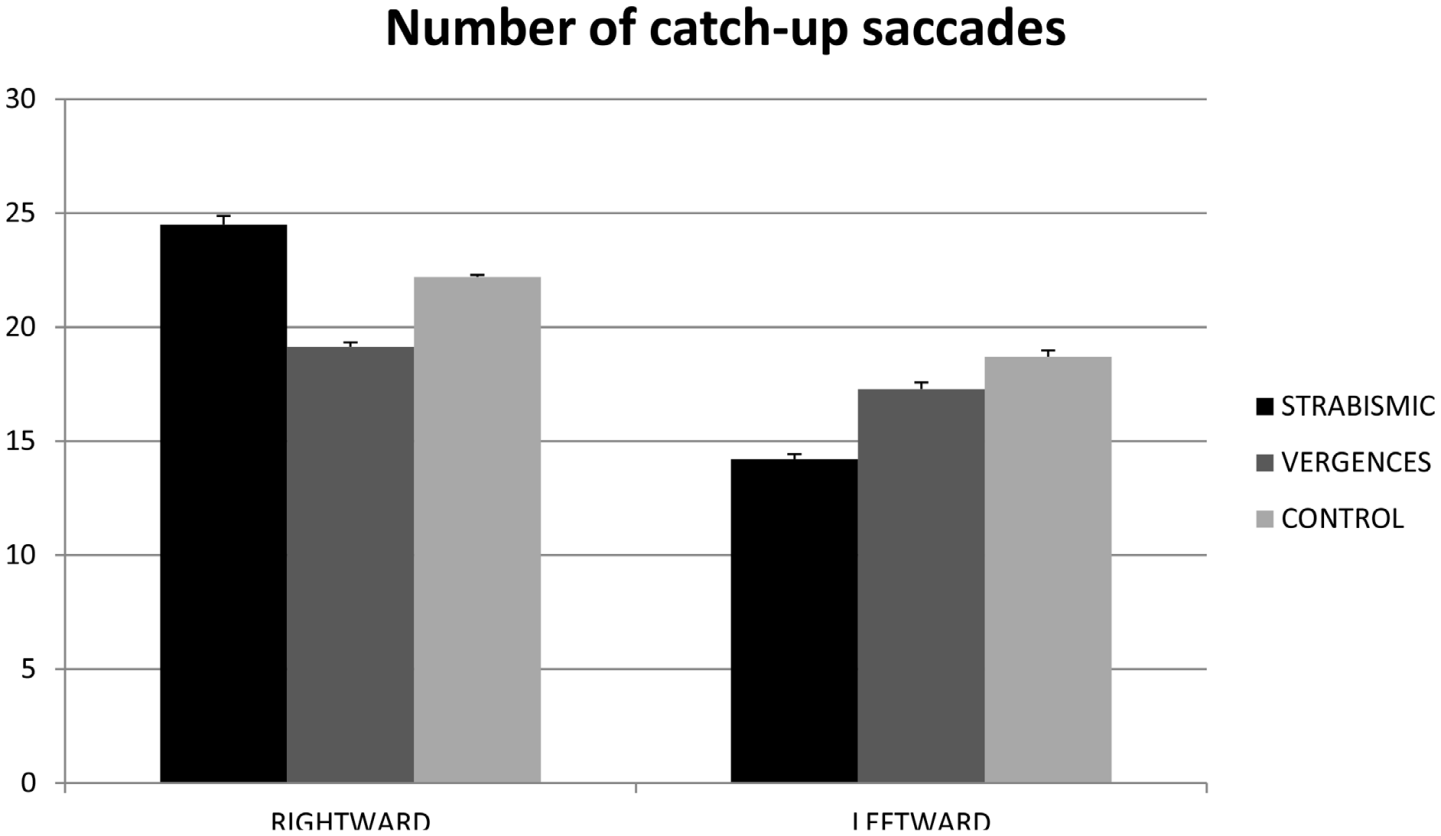
Number of the catch-up saccades. Individual mean of the number of the catch-up saccades for strabismic children, children with vergence deficits and control age-matched children. Vertical bars indicate the standard error.

Figure 2 shows the mean number of catch-up saccades in strabismic children, in children with vergence deficits, and in control age-matched children. The ANOVA test showed a significant direction effect (F_(1.21)_ = 4.08, p<0.05). The number of catch-up saccades was significantly higher in rightward than in leftward direction.

### Gain value of pursuit

**Figure 3:**
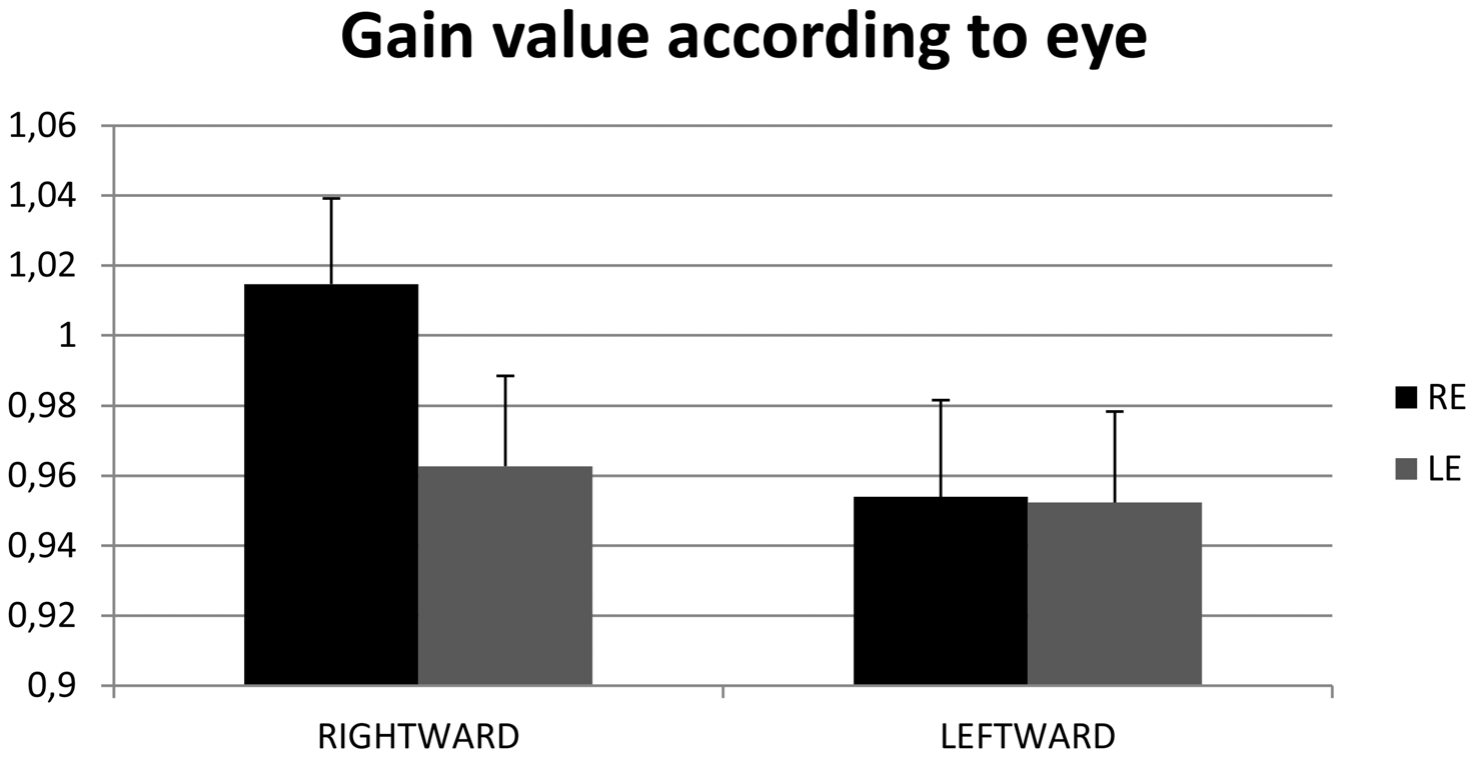
Gain value of pursuits. Individual mean of the gain value of pursuit for strabismic children, children with vergence deficits and control age-matched children. Vertical bars indicate the standard error.

Figure 3 shows the mean gain value of pursuit in strabismic children, children with vergence deficits and non-strabismic age-matched children. The ANOVA test showed a significant interaction between direction and eye (F_(1.21)_ = 7.65, p = 0.01). *Post hoc* comparisons showed that gain value in rightward direction was significantly higher in the right eye than in the left eye (p<0.02). Furthermore, gain of the right eye was significantly higher in the rightward than in the leftward direction (p<0.01).

### Disconjugacy of pursuits

**Figure 4:**
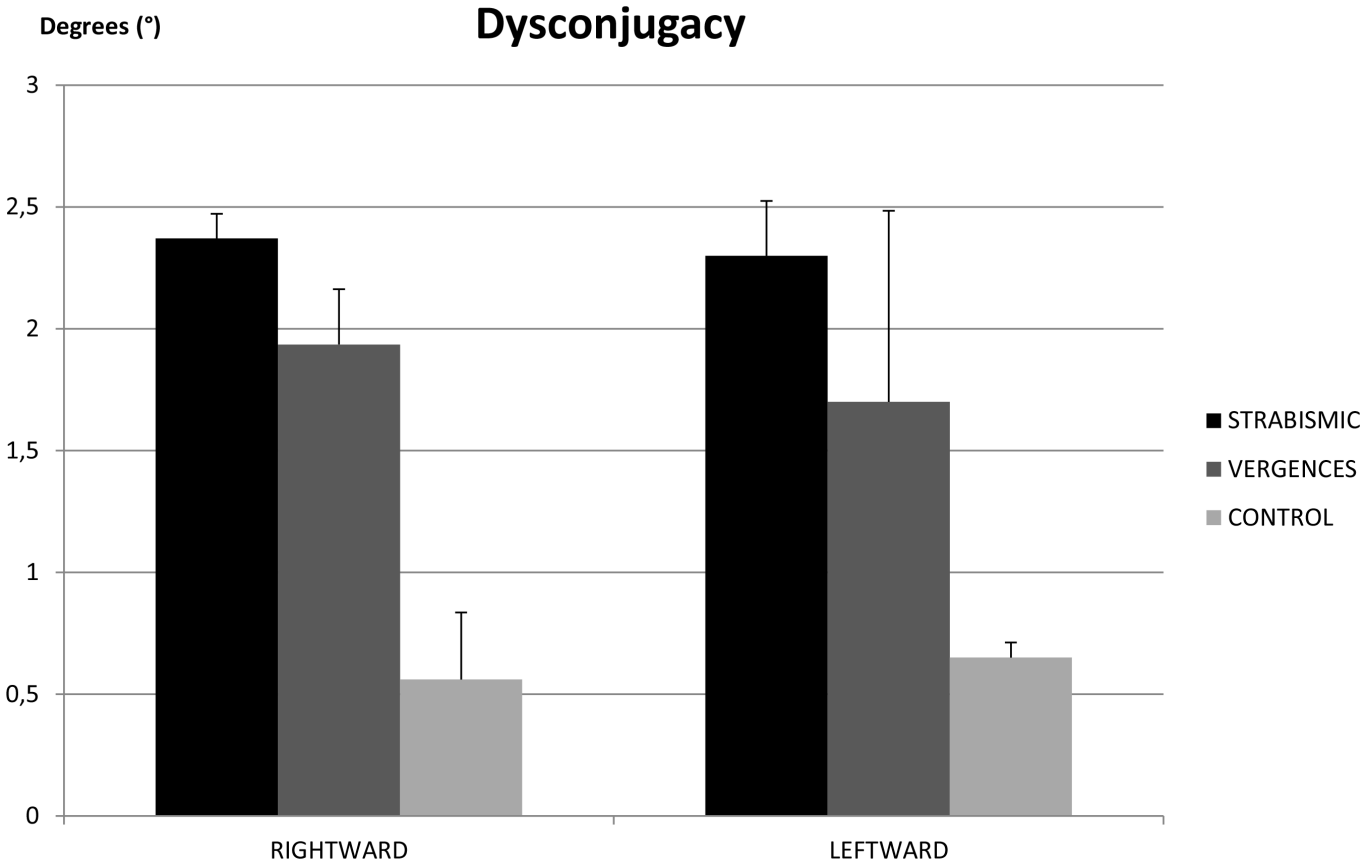
Dysconjugacy of pursuits. Individual mean of the dysconjugacy of pursuits for strabismic children, children with vergence deficits and control age-matched children. Vertical bars indicate the standard error.

Figure 4 shows the mean disconjugacy of pursuit in strabismic children, in children with vergence deficits and in control age-matched children. The ANOVA test showed a significant group effect (F_(2.24)_ = 6.30, p<0.006). *Post hoc* comparison showed that disconjugacy of pursuits in control children was significantly smaller than that observed in strabismic children (p<0.001) and in children with vergence deficits (p<0.03).

## Discussion

The main findings from this study are as follows: *(i)* the amplitude of catch-up saccades in strabismic children and in children with vergence deficits is significantly higher than in control age-matched children; in strabismic children the amplitude of catch-up saccades is significantly higher in rightward than in leftward direction; *(ii)* the number of catch-up saccades is significantly higher in rightward than in leftward direction; *(iii)* the gain value of pursuits in rightward direction is significantly higher in the right eye than in the left one; for the right eye, the gain value is significantly higher in rightward than in leftward direction; *(iv)* disconjugacy of pursuit is significantly smaller in control age-matched children than in strabismic children and in children with vergence deficits.

### Amplitude of catch-up saccades

Our study shows that the amplitude of catch-up saccades observed in strabismic children and in children with vergence deficits is significantly higher than in control age-matched children. We assume that, in order to compensate poor target position and retinal slip, strabismic children and children with vergence deficits perform larger catch-up saccades. This new finding is in line with the study of Brouwer *et al.* [19], showing that in normal adult subjects amplitude of catch-up saccades was correlated with both positional error and retinal slip. These authors advanced the hypothesis that catch-up saccades command is probably localized in cortical (middle temporal visual area, dorso-lateral pontine nuclei, and cerebellum) as well as in subcortical structures (superior colliculus).

### Number of catch-up saccades

Our findings show no difference in the number of catch-up saccades for strabismic children, children with vergence deficit or control age-matched children. This suggests that the frequency of catch-up saccades does not depend on vergence disparity capabilities or on binocular vision. This is in line with the hypothesis of Ciuffreda *et al.* [11], who found that high frequency of catch-up saccades could be related to the presence of amblyopia rather than strabismus. On the other side, as explained above, strabismic children and children with vergence insufficiency displayed larger catch-up saccades amplitudes, suggesting that pursuit performance is poorer than the one reported in control age-matched children. Based on these findings, we could advance the hypothesis that both vergence disparity and binocular vision capabilities are needed to correctly perform pursuits.

The new interesting result is that the number of catch-up saccades is significantly higher in rightward than in leftward direction. Let us recall that all children are French readers, who therefore are most accustomed to perform saccades from left to right direction during reading. This could explain the frequent number of catch-up saccades during rightward pursuit.

### Gain value of pursuits

We found that the gain value of pursuits was similar for the three groups of children tested. This suggests that the gain value of pursuit does not depend on vergence disparity capabilities or on binocular vision. This is in line with the fact that gain value depends on age and target characteristics (speed, surface area, *etc*) [20]. Several authors have shown that pursuit gain reaches the value of 0.8 at nine years old, consequently our data are in agreement with previous studies [21]–[23].

Interestingly, our data show that the gain of pursuit in rightward direction is significantly higher in the right eye than in the left eye. This has not been found previously, most likely because no studies recorded both eyes simultaneously. Moreover, the gain of the right eye is significantly higher in rightward than in leftward direction. Such direction dependency has already been reported in strabismic adult subjects, particularly for temporal direction [12]–[14].

### Disconjugacy of pursuit

Our results show that the disconjugacy of pursuit is significantly smaller in control age-matched children than in strabismic children and in children with vergence deficits. The poor quality of binocular coordination of pursuits reported here gives more insight on the quality of binocular coordination of eye movements. In the present study we show that binocular vision plays an important role in controlling binocular pursuit given that children with vergence deficits with normal binocular vision show smaller disconjugacy than strabismic children. This is in line with several previous studies of our group showing the importance of binocular vision and proper vergence disparity capabilities for a good binocular coordination of saccades [7]–[9]. Taken together, all these studies showed that the binocular coordination during and after the saccades to target-leds or saccades during reading a text was poor. The poor quality of binocular coordination of pursuit could probably be related to immaturity of normal oculomotor mechanisms responsible for fine control between the pursuit and vergence commands. These new results suggest that this relationship is deficient in strabismic children and in children with vergence deficits. Improvement in fusional capabilities, obtained by vergence orthoptic training, may improve binocular coordination of pursuits, at least in children with vergence deficits. This hypothesis, however, needs to be confirmed by further studies.
